# Fatty acid-binding protein 5 limits ILC2-mediated allergic lung inflammation in a murine asthma model

**DOI:** 10.1038/s41598-020-73935-y

**Published:** 2020-10-06

**Authors:** Shuhei Kobayashi, Shunichi Tayama, Hai The Phung, Yoshiteru Kagawa, Hirofumi Miyazaki, Yu Takahashi, Takashi Maruyama, Naoto Ishii, Yuji Owada

**Affiliations:** 1 Department of Organ Anatomy, Tohoku University Graduate School of Medicine, Sendai, 980-8575 Japan; 2 Department of Microbiology and Immunology, Tohoku University Graduate School of Medicine, Sendai, 980-8575 Japan; 3 Mucosal Immunology Unit, National Institute of Dental and Craniofacial Research, National Institutes of Health, Bethesda, MD 20895 USA; 4 Department of Immunology, Graduate School of Medicine and Faculty of Medicine, Akita University, Akita, 010-8543 Japan

**Keywords:** Immunology, Health care

## Abstract

Dietary obesity is regarded as a problem worldwide, and it has been revealed the strong linkage between obesity and allergic inflammation. Fatty acid-binding protein 5 (FABP5) is expressed in lung cells, such as alveolar epithelial cells (ECs) and alveolar macrophages, and plays an important role in infectious lung inflammation. However, we do not know precise mechanisms on how lipid metabolic change in the lung affects allergic lung inflammation. In this study, we showed that *Fabp5*^−/−^ mice exhibited a severe symptom of allergic lung inflammation. We sought to examine the role of FABP5 in the allergic lung inflammation and demonstrated that the expression of FABP5 acts as a novel positive regulator of ST2 expression in alveolar ECs to generate retinoic acid (RA) and supports the synthesis of RA from type II alveolar ECs to suppress excessive activation of innate lymphoid cell (ILC) 2 during allergic lung inflammation. Furthermore, high-fat diet (HFD)-fed mice exhibit the downregulation of FABP5 and ST2 expression in the lung tissue compared with normal diet (ND)-fed mice. These phenomena might be the reason why obese people are more susceptible to allergic lung inflammation. Thus, FABP5 is potentially a therapeutic target for treating ILC2-mediated allergic lung inflammation.

## Introduction

The prevalence of allergic lung inflammation is annually still increasing worldwide^1^, and prolonged inflammation derived from these symptoms leads to chronic diseases, such as asthma. Moreover, the development of allergic lung inflammation and asthma is strongly correlated with obesity^2,3^. It is currently not clear how allergic lung inflammation develops and whether cellular lipid metabolism involves in this process.

Innate lymphoid cells (ILCs) have been identified as a major component of the innate immune system, and that mirror helper CD4^+^ T subset, despite lacking rearranged antigen receptor^4^. Based on the cytokines they produce, ILCs are classified mainly three subsets: group 1 innate lymphoid cells (ILC1s), ILC2s, and ILC3s. Among these types of ILCs, ILC2s play pivotal roles in various types of inflammatory diseases like bronchial asthma^5^ and atopic dermatitis^6^. In the matter of allergic disease in the lung, allergen exposure drives air-way ECs into producing IL-25, IL-33, and thymic stromal lymphopoitin (TSLP). Consequently, these cytokines activate ILC2s to secrete IL-5 and IL-13, driving and/or amplifying airway hyper-reactivity^4^.

Therefore, regulatory factors that repress ILC2 function has been greatly addressed for a therapeutic target of allergic inflammation. Recently, much evidence has described the suppressive factors, such as cytokines (IL-27, IFNs)^7,8^, lipid mediators (prostaglandin I_2_, prostaglandin E_2_, lipoxinA_4_) in mouse^9^. In addition, it is also recognized that one metabolic substrate, retinoic acid (RA), suppresses ILC2 function by converting into IL-10 producing regulatory phenotype of ILC2 (ILC2reg)^10^. In humans, it is suggested that airway bronchial ECs could be the origin of RA during allergic lung inflammation^11^. However, little is known about how ECs synthesize and/or secrete RA in the setting of allergic lung inflammation.

Fatty acid-binding protein 5 (FABP5), which has a high affinity to saturated fatty acid, is expressed by various immune cells, such as macrophages, dendritic cells, and CD4^+^ T cells^12–14^. This molecule plays important roles in regulating the function of those cells through modulating gene expression and/or signal transduction^12–14^. Moreover, alveolar ECs and alveolar macrophages (AMϕ) also express FABP5 and are supposed to be helpful to maintain lung homeostasis^15–18^. Considering that the expression and function of FABP5 in the lung, it is speculated that this molecule plays a pivotal role in allergic lung inflammation, although there have been no reports about it.

In this study, we sought to determine whether FABP5 regulates allergic lung inflammation, and how FABP5 modulates the development of symptoms. Our observations provide a novel mechanism of FABP5-mediated allergic lung inflammation.

## Materials and methods

### Mice

C57BL/6J CD45.2 wild-type (WT) mice were obtained from the Japan SLC (Shizuoka, Japan). CD45.2 *Fabp5*^−/−^ mice on a C57BL/6J background have been described^19^. CD45.1 wild-type mice were kindly provided by Dr. T. Maruyama (Akita University). Mice were used for experiments at 7- to 12- weeks of age. All mice were bred and maintained under specific pathogen-free conditions. The animal protocols and procedures for experiments were approved by the Animal Committee of the Tohoku University School of Medicine Ethics Committee (2018MdLM0-022), and all experiments were conducted according to the ethical and safety guidelines of the Institute.

### Cytokine administration

Anesthetized mice were treated intranasally with 20ul of IL-33 (250 ng) (Biolegend, San Diego, CA, USA) on days 0, 1, and 2, and the mice were analyzed on day 3. For administration of retinoic acid, a total of 250 μg of all-trans-RA (Sigma-Aldrich, St. Louis, MO, USA) in 30 µl of DMSO was administered intraperitoneally to mice every day for 6 days. Three days after RA administration, RA received mice were simultaneously treated intranasally with 250 ng of IL-33 on days 4, 5, and 6, and the mice were analyzed on day 7.

### Cell preparation from the lung

To isolate hematopoietic cells from the lung, mice were perfused with 10 ml PBS. The isolated lungs were cut into several small pieces and were incubated with RPMI-1640 containing 2% FCS (v/v), 50 μg/ml Liberase TM and 10 μg/ml DNase I (both from Merck Research Laboratories) for 1 h at 37 °C. After that, all cell suspensions were passed through a 70 μm cell strainer, and then cell strainers were washed with PBS to recover cells. The total single-cell suspension was enriched by two-layer Percoll (GE, Healthcare, Buckinghamshire, UK) gradient centrifugation, and the isolated cells were used as lung hematopoietic cells.

### Flow cytometry

All cells were incubated with anti-CD16/CD32 (2.4G2) before being stained with the appropriate antibodies to cell-surface and intracellular antigens. mAbs used were directed against CD3ε (17A2), CD4 (RM4-5), CD8α (53–6.7), CD11b (M1/70), CD11c (N418), CD31(390), CD45 (30-F11), CD45R (RA3-6B2), CD45.1 (A20), CD45.2 (104), CD49 (DX5), CD326 (G8.8), FcεRIα (MAR-1), Gr1 (RB-8C5), Sca1 (D7), TCRγδ (GL3), Ter119 (TER-119) (Biolegend), ST2 (RMST2-2) (Thermo Fisher Scientific, Waltham, MA, USA). For the detection of dead cells, LIVE/DEAD Cell Stain Kit (Thermo Fisher Scientific) was used. For intracellular staining, cells were subsequently fixed with Fixation buffer and incubated with Permeabilization buffer (both BD Bioscience) supplemented with mAbs against IL-5 (TRFK5) (BioLegend) and IL-13 (eBIO13A) (Thermo Fisher Scientific). Data were acquired on a FACSCanto II (BD Bioscience, San Jose, CA, USA) and were analyzed with FlowJo software (Tree Star, Ashland, Ore, USA). Eosinophils were identified as Siglec-F^+^ CD11b^+^ cells, which were confirmed to be negative for CD11c. Type I and type II alveolar ECs were identified as CD45^-^ EpCAM^+^ CD31^-^ Pdpn^+^ I-A^b-^ and CD45^-^ EpCAM^+^ CD31^-^ Pdpn^-^ I-A^b+^, respectively. Alveolar macrophages were identified as CD45^+^ Siglec-F^+^ CD11c^+^. Endothelial cells and stromal/fibroblasts were CD45^-^ EpCAM^-^ CD31^+^ and CD45^-^ EpCAM^-^ CD31^-^, respectively. ILC2s in the lung were defined as Lin^-^ ST2^+^ cells. The lineage markers used were CD3α, CD4, CD8α, CD11b, CD11c, CD45R, CD49, FcεRIα, Gr1, TCRγδ, and Ter119.

### Immunohistochemistry

For PAS staining, lung tissues were fixed with ALTFIX (Pharma Co., Ltd., Tokyo, Japan) and embedded in paraffin. Paraffin sections were de-paraffinized using xylene, and then hydrated with water and were stained with periodic acid-Schiff to stain mucous-secreting cells. Semi-quantitative analysis of PAS scoring using the previously described method^20,21^. Mucous scores were: 0-no mucous, 1-a few cells secreting mucous, 2-many cells secreting mucous, and 3-extensive mucous production.

For other analyses, de-paraffinized sections of lung tissues were incubated overnight at 4 °C with ALDH1/2 antibody (1:50) (Santa Cruz Biotechnology, Dallas, Texas, USA). For enzyme-based immunohistochemistry, biotinylated rabbit anti-mouse IgG was used as a secondary antibody followed by reaction with 3,3′-diaminobenzidine tetrahydrochloride (Sigma-Aldrich) using VECTASTAIN Elite ABC kit (Vector Laboratories, Burlingame, CA, USA) under the manufacturer’s instructions.

### Immunofluorescence staining

For immunofluorescence staining, cultured A549 cells were washed twice with D-PBS (−) and fixed with 4% PFA. Fixed cells were permeabilized with 0.1% (v/v) Triton X-100 in PBS and blocked with 5% (v/v) normal goat serum (FUJIFILM Wako Pure Chemical Corporation, Osaka, Japan) in PBS. The reaction with primary antibodies [monoclonal anti-ST2 (1:50)] (Proteintech Group, Inc., Rosemont, IL, USA), was performed overnight at 4 °C, and the reaction with secondary antibodies [Alexa 488 anti-mouse IgG (1:500)] (Thermo Fisher Scientific) and DAPI (Thermo Fisher Scientific) was performed for 1 h at room temperature. Samples were examined by confocal laser microscopy (Zeiss AX10, Carl Zeiss, Jena, Germany).

### Generation of bone marrow chimera mice

6- to 8- week old CD45.1 WT mice were lethally irradiated (9.5 Gy) and intravenously transplanted with 50 million bone marrow cells obtained from 6- to 10- week old CD45.2 WT or *Fabp5*^−/−^ animals. Donor and recipient cells were distinguished using CD45.1/2 markers. 6 weeks after transplantation, donor cells in the lung were analyzed by flow cytometry as described above.

### RNA extraction and Real-time RT-PCR

Total RNA was extracted with RNA iso Plus (Thermo Fisher Scientific), and cDNA was then synthesized with SuperScript III Reverse Transcriptase and oligo(dT)20 (Thermo Fisher Scientific). SYBR Premix Ex Tag (Takara Bio, Kusatsu, Japan) and a 7500 real-time PCR system (Thermo Fisher Scientific) were used for quantitative RT-PCR. Each transcript was analyzed concurrently, and results were presented relative to the abundance of transcripts encoding GAPDH. Primers were shown in Table 1.

**Table 1:** Oligonucleotide primers in study.

Gene	Forward sequence	Reverse sequence
Mouse *Gapdh*	CCAGGTTGTCTCCTGCGACTT	CCTGTTGCTGTAGCCGTATTCA
Mouse *Fabp5*	TGAAAGAGCTAGGAGTAGGACTG	CTCTCGGTTTTGACCGTGATG
Mouse *Aldh1a1*	ATACTTGTCGGATTTAGGAGGCT	GGGCCTATCTTCCAAATGAACA
Mouse *Aldh1a2*	CAGAGAGTGGGAGAGTGTTCC	CACACAGAACCAAGAGAGAAGG
Mouse *Pparγ*	TGTGGGGATAAAGCATCAGGC	CCGGCAGTTAAGATCACACCTA
Mouse *St2*	TGTATTTGACAGTTACGGAGGGC	ACTTCAGACGATCTCTTGAGACA
Mouse *Il5*	ACAAGCAATGAGACGATGAGGC	TTTCCACAGTACCCCCACGG
Mouse *Il13*	CTCCCTCTGACCCTTAAGGAGCTT	GGTCCACACTCCATACCATGCTG
Human *Gapdh*	GGAGCGAGATCCCTCCAAAAT	GGCTGTTGTCATACTTCTCATGG
Human *Fabp5*	TGAAGGAGCTAGGAGTGGGAA	TGCACCATCTGTAAAGTTGCAG
Human *Aldh1a1*	GCACGCCAGACTTACCTGTC	CCTCCTCAGTTGCAGGATTAAAG
Human *Aldh1a2*	TTGCAGGGCGTCATCAAAAC	ACACTCCAATGGGTTCATGTC
Human *Pparγ*	GGGATCAGCTCCGTGGATCT	TGCACTTTGGTACTCTTGAAGTT
Human *St2*	ATGGGGTTTTGGATCTTAGCAAT	CACGGTGTAACTAGGTTTTCCTT

### Cell culture and FABP5-knockdown

Human alveolar epithelial cells line A549 was obtained from Tohoku University for Cell Resource Center for Biomedical Research. A549 cells were maintained by a passage in DMEM medium (FUJIFILM Wako Pure Chemical Corporation) supplemented with 10% heat-inactivated fetal bovine serum (Thermo Fisher Scientific), 100 U/ml penicillin, 100 μg/ml streptomycin (Sigma-Aldrich), and 2 mmol/l l-glutamine (Thermo Fisher Scientific). A549 cells were transfected with either E-FABP siRNA or Control siRNA by using siRNA Transfection Reagent (Santa Cruz Biotechnology, Inc., Dallas, TX, USA). For transient knockdown, cells were used 48 h after transfection and stimulated recombinant human IL-33 (50 ng/ml) (Biolegend) for 24 h.

### HFD-induced obesity

6-Week old male mice were fed either Normal Diet (ND) (D12450, 3.85 kcal/g, 10% of energy as fat; Research Diets, New Brunswick, NJ, USA) or High-Fat Diet (HFD) (D12492, 5.24 kcal/g, 60% of energy as fat; Research Diets). Ten weeks later, ND- or HFD-fed mice were sacrificed and analyzed.

### Statistical analysis

Statistical analyses were performed using Student’s t-test (two-tailed), and one-way analysis of variance was used to test differences between groups for continuous variables. *p < 0.05, **p < 0.01, ***p < 0.001.

## Results

### FABP5 deficient mice display more severe allergic lung inflammation

**Figure 1:**
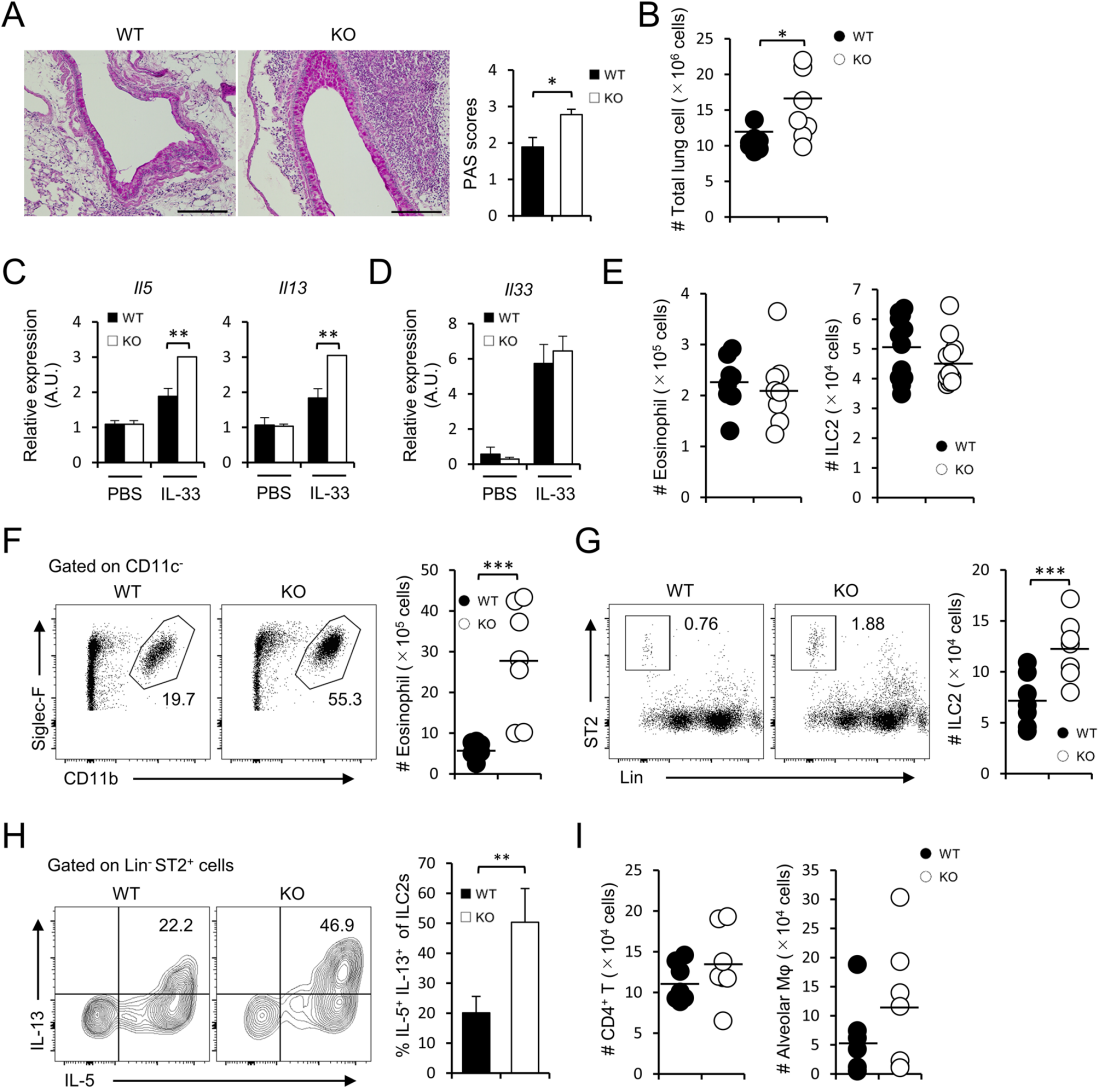
FABP5 limits allergic lung inflammation. (**A–I**) IL-33-injected wild-type (WT) and *Fabp5*^*−/−*^ (KO) mice. (**A**) Representative PAS lung sections and scoring graph (n = 8). Scale bar, 200 μm. (**B**) The number of total lung cells in the lung. (**C**,** D**) Expression of *Il5*, *Il13* (**C**), and *Il33* (**D**) of whole lung tissues from IL-33 administrated mice. (**E**) The absolute number of eosinophils and ILC2s in the lung from PBS-treated control mice. (**F**–**H**) The absolute number of eosinophils (**F**), ILC2s (**G**), and CD4^+^ T cells, alveolar macrophages (**H**) in the lung. Numbers adjacent to the outline areas indicate the percentage of eosinophils (Siglec-F^+^CD11b^+^) and ILC2s (Lin ^−^ST2^+^). (**I**) The frequency of IL-5^+^IL-13^+^ cells among lung ILC2s. *p < 0.05, **p < 0.01, and ***p < 0.001. Data are from one experiment representative of three independent experiments with similar results (**A**,** C**,** D**,** I**) or are from three pooled (**B**,** E–H**) experiments.

FABP5 is involved in the pathogenesis of viral/bacterial lung infection and the airway inflammation in allergic asthmatics^15,16,22^. To evaluate the precise role of FABP5 in allergic lung inflammation, we intranasally administrated recombinant IL-33, one of the cytokines known to cause lung inflammation through ILC2 activation, into mice and examined lung inflammation in wild-type and *Fabp5*^−/−^ mice. PAS staining of mucus in IL-33-treated lungs showed a higher amount of mucus production from airway goblet cells in the lungs of *Fabp5*^−/−^ mice when compared to those of wild-type mice (Fig. 1A). The total number of cells infiltrating into lung tissues also was significantly increased in *Fabp5*^−/−^ mice (Fig. 1B). Consistent with the results of the cell infiltration, *Il-5* and *Il-13* expression in the lung tissues from *Fabp5*^−/−^ mice markedly enhanced under the inflammatory condition, while the expression of *Il-33* was no difference between the two groups (Fig. 1C,D). Although the numbers of eosinophils and ILC2s were comparable under steady-state conditions, IL-33-treated *Fabp5*^−/−^ mice displayed an augmented inflammation with massive infiltration of these cells in the lung tissues (Fig. 1E–G). Furthermore, we observed that IL-33-treated *Fabp5*^−/−^ mice also showed a higher frequency of IL-5^+^IL-13^+^ILC2s when compared to wild-type mice (Fig. 1H). In contrast to the increased eosinophils and ILC2s, CD4^+^ T cells and alveolar macrophages were not affected in *Fabp5*^−/−^ mice (Fig. 1I). These results suggest that FABP5 negatively regulates ILC2-mediated allergic lung inflammation.

### FABP5 deficiency in non-hematopoietic cells is crucial for the pathogenesis of allergic lung inflammation

**Figure 2:**
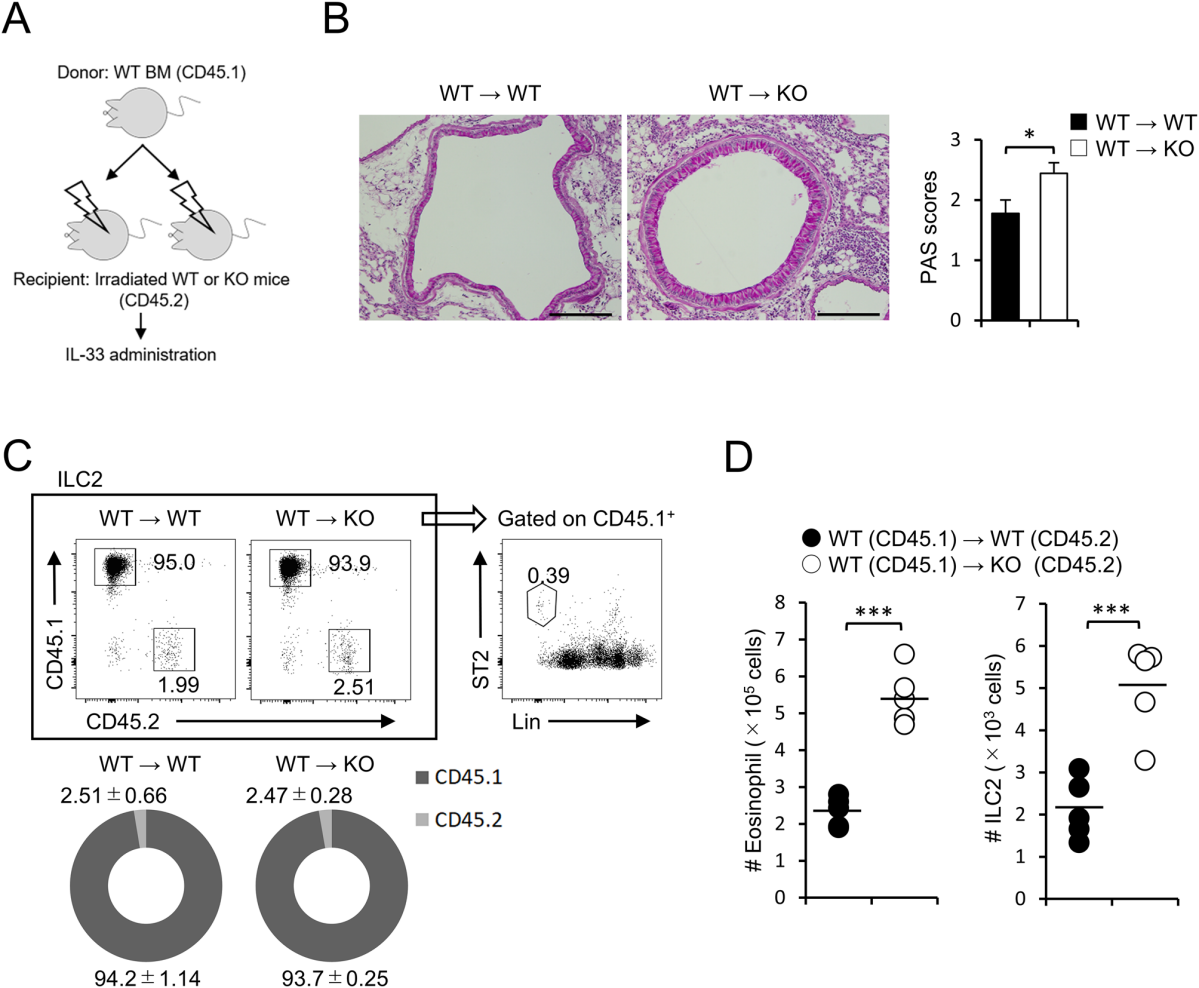
FABP5 in non-hematopoietic cells is important for ILC2-mediated lung inflammation. (**A–D**) IL-33-injected bone chimera WT or KO recipient mice. (**A**) Schematic diagram of a bone marrow transplantation model. (**B**) Representative PAS lung sections and scoring graph (n = 6). Scale bar, 200 μm. (**C**) The frequency of chimerism in the lung from bone marrow transplanted recipient mice. (**D**) The absolute number of eosinophils and ILC2s derived from CD45.1^+^ donor cells in the lung. ***p < 0.001. Data are from one experiment representative of at least three independent experiments with similar results (**B**) or are from three pooled (**C**, **D**) experiments.

Although lung Alveolar epithelial cells, macrophages and airway epithelial cells express FABP5^15^, most of the cells that constitute lung tissues are alveolar epithelial cells. In addition, we determined lung ILC2s slightly express FABP5 (data not shown). To evaluate which FABP5 expression in non-hematopoietic cells or ILC2s are responsible for severe symptom of allergic lung inflammation, we performed a bone marrow transplantation experiment, in which donor bone marrow donor cells from congenic wild-type mice (CD45.1) were transplanted into lethally irradiated wild-type (CD45.2) or *Fabp5*^−/−^ (CD45.2) recipient mice (Fig. 2A). Next, we treated the recipient mice with an intranasal administration of IL-33 before assessing the condition of lung inflammation. Consistent with results using wild-type and *Fabp5*^−/−^ mice, *Fabp5*^−/−^ recipient mice showed high levels of cell infiltration and mucus production (Fig. 2B). Since it is known that ILC2s in some tissues are radioresistant, we next confirmed the ratio of chimerism in the lung from the recipient mice. In the case of our results, we observed that lung ILC2s were replaced with a high proportion of CD45.1^+^ donor cells (Fig. 2C). Additionally, ILC2s and eosinophils derived from CD45.1^+^ donor cells were significantly infiltrated in the lung of *Fabp5*^−/−^ recipient mice (Fig. 2D), indicating that FABP5 deficiency in non-hematopoietic cells plays a dominant role in the exacerbated allergic lung inflammation.

### Retinoic acid administration attenuates the severe inflammation in *Fabp5*^−/−^ mice

**Figure 3:**
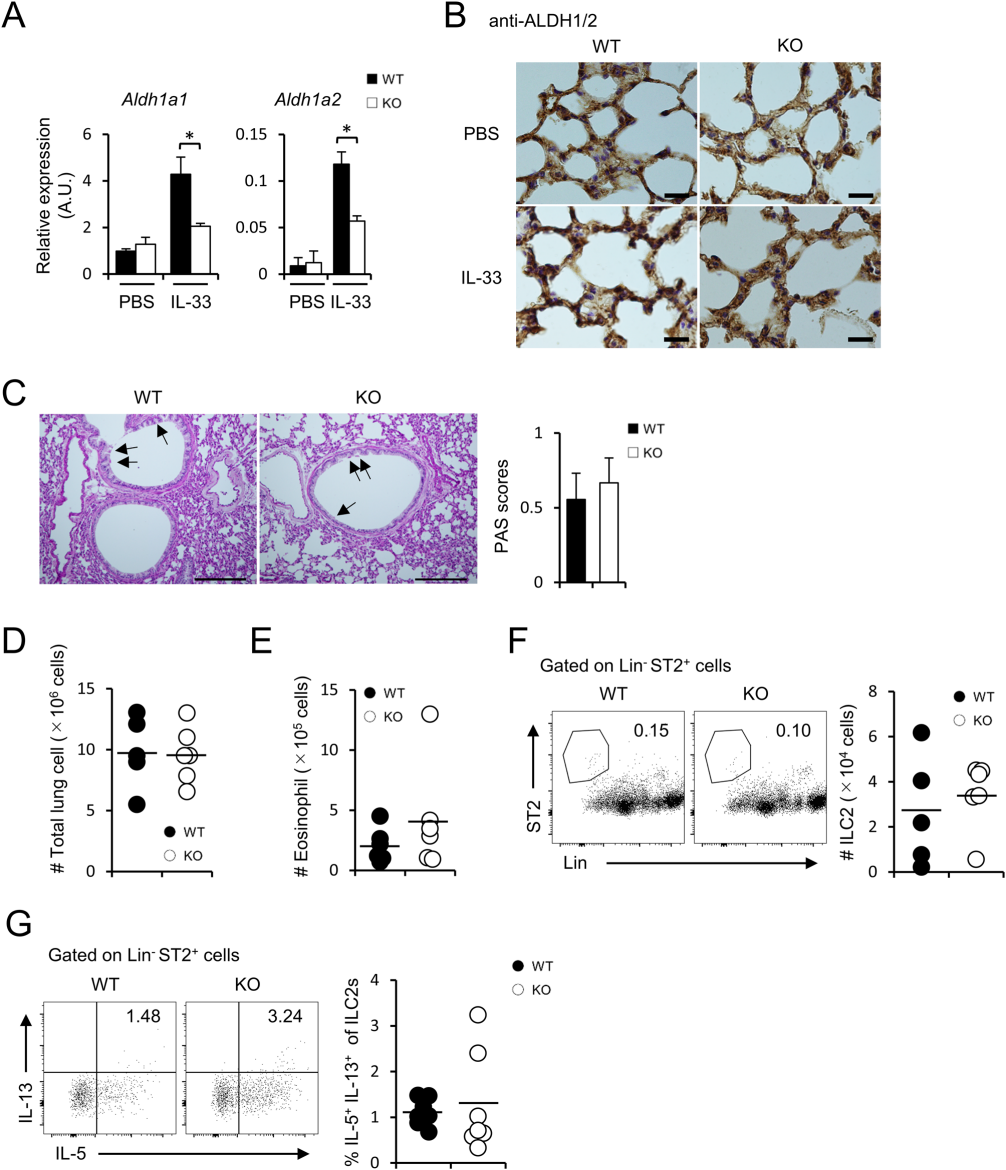
Retinoic acid treatment leads to attenuation of ILC2-mediated allergic lung inflammation. (**A**) Expression of *Aldh1a1*, *Aldh1a2* mRNA of whole lung tissues from IL-33 administrated mice. (**B**) Representative DAB staining of ALDH1/2 in lung section (n = 9). scale bar, 20 μm. (**C**) Representative PAS lung sections and scoring graph (n = 5). Scale bar, 200 μm. (**D**) The number of total lung cells in the lung from RA and IL-33-administrated mice. (**E**, **F**) The absolute number of eosinophils (**E**), and ILC2s (**F**) in the lung. Numbers adjacent to the outline areas indicate the percentage of ILC2s (Lin^−^ST2^+^). (**G**) The frequency of IL-5^+^IL-13^+^ cells among lung ILC2s in the lung from RA and IL-33-administrated mice. *p < 0.05. Data are from one experiment representative of three independent experiments with similar results (**A–C**) or are from three pooled (**D–G**) experiments. [mean ± S.E.M. of three replicates (**A**)].

Retinoic acid (RA), a vitamin A metabolite, induces alveolar regeneration^23^, and contributes to the maintenance of lung homeostasis, whereas RA administration leads to a reduction of IL-13 producing ILC2 number^11,24^. Considering this knowledge, we hypothesized that the expression of RA in *Fabp5*^−/−^ lung tissue was changed. We found that IL-33 treated *Fabp5*^−/−^ mice showed impaired expression of *Aldh1a1* and *Aldh1a2*, which are retinaldehyde dehydrogenases (RA synthesis enzyme), in whole lung tissues (Fig. 3A,B).

To next confirm the effect of RA in allergic lung inflammation, we intraperitoneally treated RA into wild-type and *Fabp5*^−/−^ mice, then these mice were further administrated intranasally with IL-33 to evaluate allergic lung inflammation. PAS staining of the lung tissues showed a decrease of mucus production compared with IL-33 alone administration, and no change of mucus production between the RA treated wild-type and *Fabp5*^−/−^ mice (Fig. 3C). Consistent with the PAS staining analysis, the numbers of lung total cells, eosinophils, and ILC2s were also comparable between the two groups (Fig. 3D–F). Furthermore, RA-treatment normalized an increasing frequency of IL-5^+^IL-13^+^ILC2s observed in *Fabp5*^−/−^ mice (Fig. 3G). These results suggest reduced RA production in the lungs of *Fabp5*^−/−^ mice might be unable to suppress ILC2 activation and lead to exacerbated allergic lung inflammation in those mice.

### FABP5 in alveolar epithelial cells is responsible for RA synthesis during allergic lung inflammation

**Figure 4:**
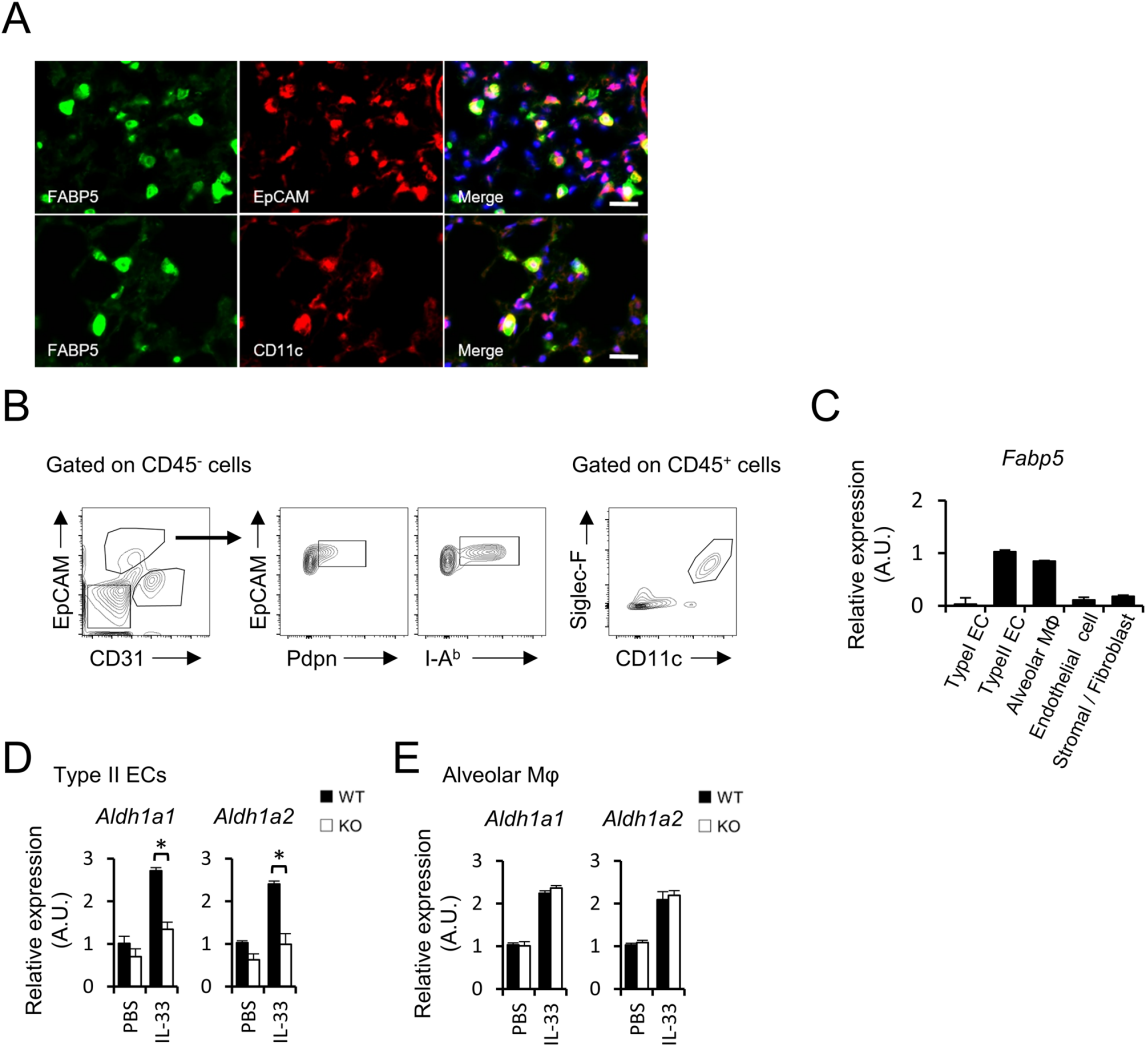
Type II alveolar epithelial cells express FABP5. (**A**) Immunostaining of lung tissue for FABP5 and EpCAM or CD11c. scale bar, 20 μm. (**B**) Gating strategy of type I alveolar ECs (CD45^−^CD31^−^EpCAM^+^Pdpn^+^), type II alveolar ECs (CD45^−^CD31^−^EpCAM^+^I-A^b+^), alveolar macrophage (CD45^+^CD11c^+^Siglec-F^+^), endothelial cell (CD45^-^ CD31^+^EpCAM^−^) and stromal cell/fibroblast (CD45^−^CD31^−^EpCAM^−^). (**C**) Expression of *Fabp5* mRNA of isolated lung cells from naive WT mice. (**D**,** E**) Expression of *Aldh1a1* and *Aldh1a2* mRNA of isolated type II alveolar ECs (**D**) and alveolar macrophages (**E**) from IL-33 administrated mice. *p < 0.05. Data are from one experiment representative of three independent experiments with similar results (**A**–**E**). [mean ± S.E.M. of three replicates (**C**–**E**)].

As mentioned above, FABP5 is expressed on a variety of lung cells^15–17^. Immunostaining with EpCAM or CD11c and FABP5 showed that the FABP5^+^ cells overlapped with the EpCAM^+^ or CD11c^+^ cells in lung tissue (Fig. 4A). To further examine this point, we evaluated FABP5 expression in isolated lung cells (Fig. 4B). As previously reported^25^, type II alveolar ECs and alveolar macrophages strongly expressed FABP5, whereas type I ECs, endothelial cell and stromal/fibroblast showed low levels of expression (Fig. 4C). Furthermore, we found that type II alveolar ECs from IL-33-administrated *Fabp5*^−/−^ mice failed to upregulate *Aldh1a1* and *Aldh1a2* expression (Fig. 4D), while alveolar macrophages from same mice expressed these genes comparable to those from wild-type mice (Fig. 4E). These results suggest that type II alveolar ECs are a major source of RA production and an important regulator of allergic lung inflammation in this model.

### FABP5 regulates ST2 expression in alveolar epithelial cells

**Figure 5:**
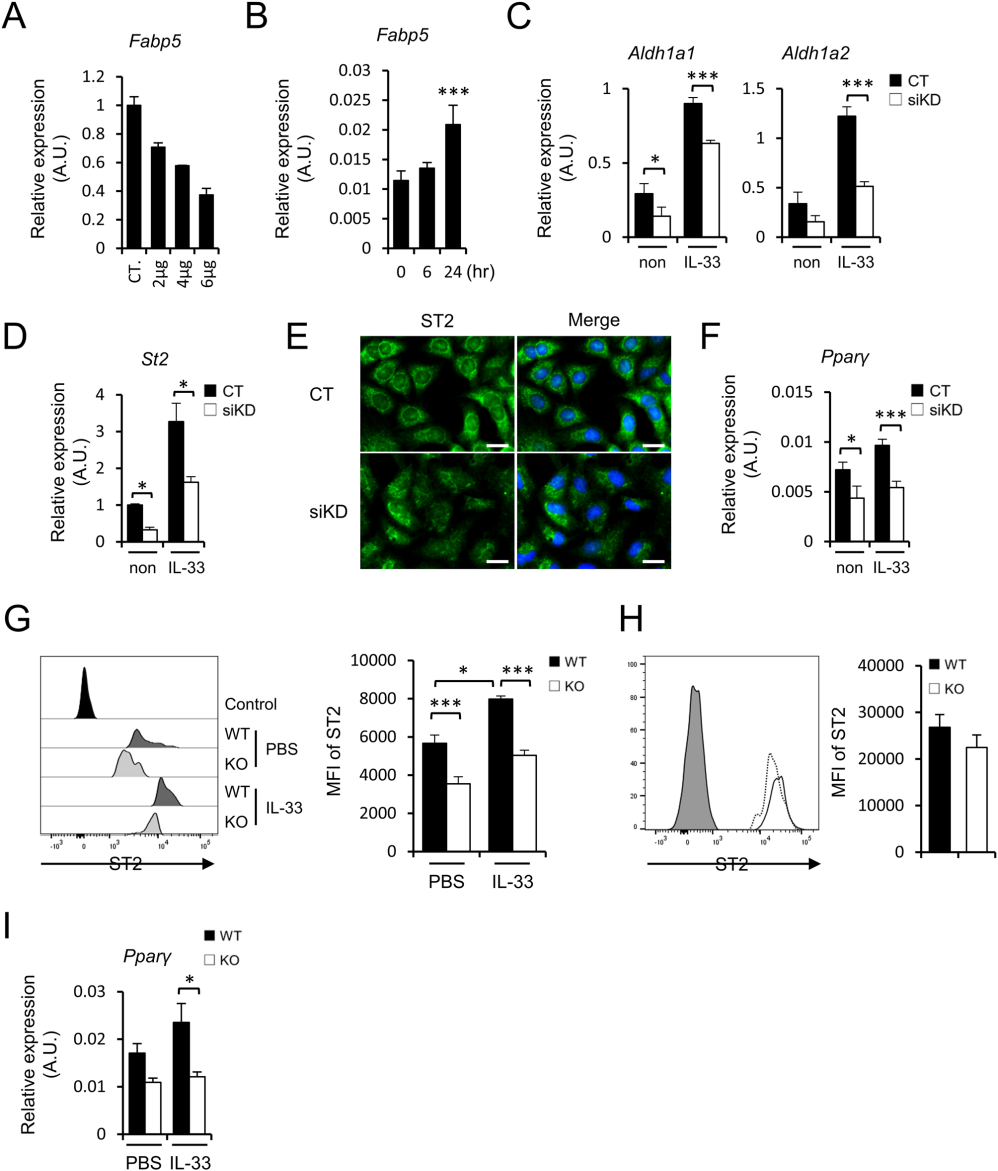
FABP5-knockdown alveolar epithelial cells reduced expression of ST2. (**A**,** B**) Expression of *Fabp5* mRNAs in A549 cells using knockdown system (**A**) and in A549 cells stimulated with IL-33 at indicated times (**B**). (**C**,** D**) Expression of *Aldh1a1*, *Aldh1a2* (**C**) and *St2* (**D**) of A549 cells stimulated with IL-33. (**E**) Immunofluorescence staining of ST2 (green) and DAPI (blue) in A549 cells. Scale bar: 50 μm. (**F**) Expression of *Pparγ* of A549 cells stimulated with IL-33. (**G**,** H**) Representative histogram displaying expression of ST2 on type II alveolar epithelial cells (**G**) and ILC2s (**H**) from WT or KO mice and the bar graph showing the corresponding mean fluorescence intensity (MFI) (mean ± S.E.M) are depicted (n = 8—11). (**I**) Expression of *Pparγ* mRNA of isolated type II alveolar ECs from IL-33 administrated mice. Data shown are representative of three independent experiments. *p < 0.05 and ***p < 0.001. Data are from one experiment representative of three independent experiments with similar results [mean ± S.E.M. of three replicates (**A**–**D**,** F**–**I**)].

Alveolar epithelial cells play important roles in lung homeostasis^26,27^. To clarify the mechanism of how the expressions of *Aldh1a1* and *Aldh1a2* were decreased in FABP5-deficiency, we conducted a knockdown system using A549 cells, a human lung alveolar epithelial cell line. We first confirmed the efficiency of *Fabp5* gene knockdown (Fig. 5A). We then found that IL-33-stimulated A549 cells upregulate FABP5 expression (Fig. 5B). In addition, FABP5-knockdown A549 cells displayed decreased expression of *Aldh1a1* and *Aldh1a2,* and failed to upregulate these genes in response to IL-33 stimulation (Fig. 5C). To examine the underlying mechanism, we evaluated ST2 expression, a receptor for IL-33, on A549 cells. We found that ST2 expression also decreased on FABP5-knockdown A549 cells and was impaired in upregulation in response to IL-33 stimulation when compared to control cells (Fig. 5D). Consistent with qPCR results, fluorescent immunostaining confirmed that ST2 expression is downregulated by FABP5 knockdown (Fig. 5E). On the other hand, It was reported that peroxisome proliferator-activated receptor (PPAR)γ, which is activated by FABP5, is known to induce retinal dehydrogenase and is attributed to the increment of the intracellular synthesis of all-trans retinoic acid (ATRA) from retinol^28^. FABP5-knockdown A549 cells impaired expression of *Pparγ* (Fig. 5F).

In line with the findings of A549 cells, we found that *Fabp5*^−/−^ type II alveolar ECs, but not lung ILC2s, exhibited a lower level of ST2 expression compared with wild-type (Fig. 5G,H). Furthermore, IL-33-challenged *Fabp5*^−/−^ type II alveolar ECs showed impaired upregulation of ST2 (Fig. 5G). Type II alveolar ECs from IL-33-administrated *Fabp5*^−/−^ mice failed to upregulate *Pparγ* expression (Fig. 5I), These results suggest that FABP5 in alveolar epithelial cells is important for lung homeostasis during IL-33-induced allergic lung inflammation.

### High fat diet-fed mice exhibit a phenotype similar to the lungs of FABP5-deficient mice

**Figure 6:**
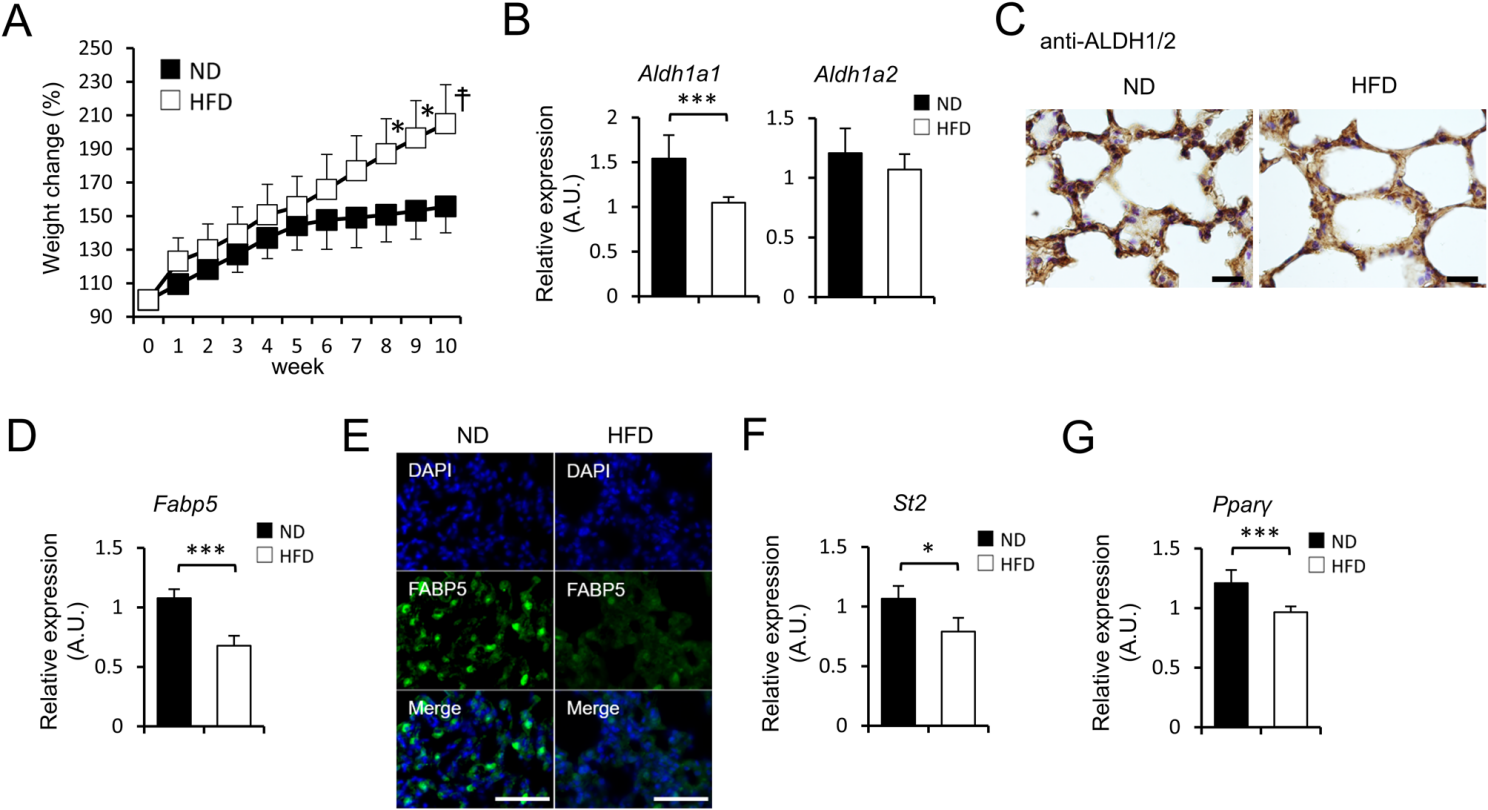
HFD-fed mice exhibited downregulation of FABP5 and ST2 expression in the lung. (**A**) Body weights of WT mice treated with ND or HFD since 6 weeks of age (n = 5–8). *p < 0.05 and ^†^*p* < 0.01 between ND and HFD. (**B**,** C**) Expression of *Aldh1a1*, *Aldh1a2* mRNA (**B**) and ALDH1/2 DAB staining (**C**) of whole lung tissues from ND- or HFD-fed mice. (**D**,** E**) Expression of *Fabp5* mRNA (**D**) and immunefluorescence staining (**E**) of whole lung tissues from ND- or HFD-fed mice. (**F**,** G**) Expression of *St2* (**F**) and *Pparγ* (**G**) of whole lung tissues from ND- or HFD-fed mice. *p < 0.05 and ***p < 0.001. Data are from three pooled (**A**) experiments and from one experiment representative of three independent experiments with similar results (**B**–**G**). (mean ± S.E.M. of three replicates (**B**,** D**,** F**,** G**).

HFD-induced obesity leads to exacerbation of allergic lung inflammation^29,30^. Moreover, HFD and genetic models of obesity demonstrated decreased tissue levels of vitamin A^31^. Considering these reports, we hypothesized that HFD-treated mice illustrated an impaired suppression of ILC2 during allergic lung inflammation due to the reduction of retinaldehyde dehydrogenase expression in lung tissue. To test this hypothesis, we analyzed *Aldh1a1* and *Aldh1a2* expression of the lungs derived from HFD-treated mice (Fig. 6A), and found that there were decreased levels of lung *Aldh1a1* and *Aldh1a2* in HFD-treated mice when compared with lean ND-fed mice (Fig. 6B,C). Interestingly, HFD-treated mice also exhibited decreased expressions of *Fabp5*, *St2,* and *Pparγ* in the lung tissues compared with ND-fed mice (Fig. 6D–G), suggesting a similar phenomenon observed in *Fabp5*^−/−^ mice. These suggest that the lipid environment change in the lung tissue caused by HFD administration might reduce the expression of FABP5 and leads to the exacerbation of allergic lung inflammation in obesity.

## Discussion

In this study, we addressed whether FABP5 plays an important role in allergic lung inflammation, and our results revealed that FABP5 positively regulates ST2 expression in alveolar ECs to express retinaldehyde dehydrogenases, contributing to an optimal inflammation during ILC2-mediated allergic lung inflammation.

Epidemiological studies have been shown that obesity is linked to a wide range of respiratory diseases, such as allergic lung inflammation^2,3^. Obesity is a complex biological condition that affects physiological responses, such as the immune system^32,33^. There's a possibility that changes in these physiological responses by comorbidities have a dramatic effect on the response to inflammatory stimuli in the lung^34^. This has made it difficult to attribute the increase in adipose tissue mass due to obesity to a change in the lung immune response in the disease setting. It is likely that obesity and its comorbidities collectively alter the immune response in the lungs, increasing susceptibility to allergic lung inflammation. FABP5 is known to play a role in the onset of obesity by regulating several obesity-related metabolisms^35,36^. Besides, this molecule is reported to associate with allergic lung inflammation^22^. However, the mechanism by which FABP5 is involved in allergic lung inflammation remains poorly understood. Additionally, there are also few reports of single factors linking obesity to the pathogenesis of allergic lung inflammation. Therefore, we focused on FABP5, which preferentially bind to saturated fatty acids, and evaluated the relationship between lipid metabolism and lung inflammation using mice with physiologically altered intracellular lipid metabolism. In this study, FABP5 deficiency significantly worsened IL-33-induced ILC2-mediated allergic lung inflammation in mice. Interestingly, the results of bone marrow transplantation showed that the expression of FABP5 in non-hematopoietic cells, but not in hematopoietic cells, regulates the symptoms of ILC2-mediated allergic lung inflammation. Additionally, our results reveal that FABP5 in alveolar ECs is important for these phenomena. However, there is no report showing the effects of cellular lipid metabolism on alveolar ECs.

FABP5 represents a key transport protein of long-chain fatty acid and is involved in the regulation of biological reactions mediated by activation of PPAR^37,38^. The previous report showed PPARγ agonist stimulation decreased allergic lung inflammation^39^. Another report also showed that PPARγ agonist stimulation induced retinaldehyde dehydrogenases^40^, suggesting that PPARγ is likely to directly target retinaldehyde dehydrogenases. Thus, our observation of reduced retinaldehyde dehydrogenases expression in the lung of *Fabp5*^−/−^ mice could be a consequence of the insufficient level of PPARγ in FABP5-deficient alveolar ECs. Another possibility is that attenuation of IL-33 signaling caused by ST2 downregulation. Our results revealed that IL-33 treatment upregulated retinaldehyde dehydrogenases in wild-type mice or si-control-A549 cells, suggesting that IL-33 signaling might promote expression of retinaldehyde dehydrogenases in alveolar ECs. However, FABP5-deficiency or knockdown-alveolar ECs displayed lower expression of ST2, and IL-33 signaling was attenuated. It was considered that this down-regulation of ST2 was attributed to the decrease in the expression of PPARγ by FABP5-deficiency or knockdown, since PPARγ is involved in ST2 regulation^41^.

Retinoic acid, a metabolite of vitamin A, is known to be involved in maintaining homeostasis of epithelial and mucosal tissues and regulating immune responses^42–44^. Although it has been reported that vitamin A supplementation induces aggravation of asthma in a mouse experimental model, the fact that RA suppresses allergic lung diseases, such as asthma is now widely accepted. In line with this, in humans, vitamin A deficiency is also associated with an increased incidence of infantile asthma accompanied by lung mucosal damage and disruption of airway epithelial maintenance^45^. Additionally, the administration of RA inhibits the activation of IL-5 and IL-13 producing ILC2 or enhancing the induction of Treg^24,46^. Our results revealed an impaired expression of retinaldehyde dehydrogenases in the lung tissue of *Fabp5*^−/−^ mice or FABP5-knockdown alveolar ECs. Thus we hypothesized that inadequate RA could not fully control the activation of ILC2 during inflammation, which might explain why *Fabp5*^−/−^ mice exhibited a severe symptom of IL-33-induced allergic lung inflammation comparison with the wild-type mice.

Furthermore, HFD-induced obesity causes the downregulation of FABP5 and retinaldehyde dehydrogenases in the lung tissue of mice. Obesity was reported to lead to vitamin A deficiency in some tissues including lung^31^, and because of this, *Fabp5*^−/−^ mice may be exhibited exacerbation of ILC2-mediated allergic lung inflammation.

FABPs are involved in epigenetic regulation in cortical neurons^47^. Epigenetic regulation, including chromatin status, DNA methylation, and histone modification, is induced by dietary intake of fatty acid, which affects gene expression^48–50^. It is considered that this is because dietary fatty acids cause a change in mitochondrial function. Besides, mitochondrial dysfunction has been reported to be involved in gene expression^51^. Furthermore, a recent study revealed that FABP5 regulates mitochondrial functions such as OXOPHOS, lipid metabolism, and maintenance of cristae structure^52^. Considering these findings, it is possible that mitochondrial function in alveolar ECs affected by intracellular lipid metabolism probably controls their ST2 gene expression. Future studies will reveal how physiological lipid metabolism regulates ST2 expression and maintenance in a homeostatic environment.

In conclusion, we have revealed a novel function of FABP5 in ILC2-mediated allergic lung inflammation via regulating the expression of ST2 receptor in alveolar ECs. This study provides a new therapeutic approach for a wide range of allergic diseases associated with ILC2 activation.

## Data Availability

The authors confirm that the data supporting the findings of this study are available within the article.

## Abbreviations

EC: Epithelial cell
FABP: Fatty acid-binding protein
HFD: High fat diet
ILC: Innate lymphoid cell
ND: Normal diet
PPARγ: Peroxisome proliferator-activated receptor γ
RA: Retinoic acid
